# A practical method to screen and identify functioning biomarkers in nasopharyngeal carcinoma

**DOI:** 10.1038/s41598-021-86809-8

**Published:** 2021-03-31

**Authors:** Chengyou Liu, Peijie Guo, Leilei Zhou, Yuhe Wang, Shuchang Tian, Yong Ding, Jing Wu, Junlin Zhu, Yu Wang

**Affiliations:** 1 Department of Medical Engineering, Nanjing First Hospital, Nanjing Medical University, 68 Changle road, Nanjing, 210006 Jiangsu Province China; 2 Department of Functional Examination, Nanjing First Hospital, Nanjing Medical University, 68 Changle road, Nanjing, 210006 Jiangsu Province China; 3 Department of Medical Imaging, Nanjing First Hospital, Nanjing Medical University, 68 Changle road, Nanjing, 210006 Jiangsu Province China; 4 Department of Mathematics and Computer, Nanjing Medical University, 140 Hanzhong road, Nanjing, 210006 Jiangsu Province China; 5 Department of Critical Care Medicine, Nanjing First Hospital, Nanjing Medical University, 68 Changle road, Nanjing, 210006 Jiangsu Province China; 6 Department of Medical Affairs, Nanjing First Hospital, Nanjing Medical University, 68 Changle road, Nanjing, 210006 Jiangsu Province China

**Keywords:** Cancer, Computational biology and bioinformatics, Genetics, Molecular biology

## Abstract

Nasopharyngeal carcinoma (NPC) is a rare malignancy, with the unique geographical and ethnically characteristics of distribution. Gene chip and bioinformatics have been employed to reveal regulatory mechanisms in current functional genomics. However, a practical solution addressing the unresolved aspects of microarray data processing and analysis have been long pursuit. This study developed a new method to improve the accuracy of identifying key biomarkers, namely Unit Gamma Measurement (UGM), accounting for multiple hypotheses test statistics distribution, which could reduce the dependency problem. Three mRNA expression profile of NPC were selected to feed UGM. Differentially expressed genes (DEGs) were identified with UGM and hub genes were derived from them to explore their association with NPC using functional enrichment and pathway analysis. 47 potential DEGs were identified by UGM from the 3 selected datasets, and affluent in cysteine-type endopeptidase inhibitor activity, cilium movement, extracellular exosome etc. also participate in ECM-receptor interaction, chemical carcinogenesis, TNF signaling pathway, small cell lung cancer and mismatch repair pathway. Down-regulation of CAPS and WFDC2 can prolongation of the overall survival periods in the patients. ARMC4, SERPINB3, MUC4 etc. have a close relationship with NPC. The UGM is a practical method to identify NPC-associated genes and biomarkers.

## Introduction

Nasopharyngeal carcinoma (NPC) is one kind of cancer that occurs in the nasopharynx, and is located behind nose and above the back of throat. Although it is not common among population, NPC has high incidence in some regions and ethnicities, especially in southern China, North Africa, and Southeast Asia^1^. In Guangdong Province, NPC accounts for 18% of all cancer in the population^2^. In 2018, approximately 73,000 deaths and 129,000 new cases were claimed by this disease globally. Signs and symptoms related to the primary tumor include trismus, pain, otitis media, and nasal regurgitation due to paresis (lost or impaired movement) of the soft palate, hearing loss and cranial nerve palsy (paralysis). The growth of disease loci may lead to nasal obstruction or bleeding and a "nasal twang". Metastatic spread may result in bone pain or organ dysfunction.

Apart from the established risk factors such as viral, dietary and genetic factors, smoking, alcohol intake, and consumption of certain pickled foods also increase the risk of NPC, which accounts for the higher incidence in males and geographical distinctive distribution of NPC^3^. NPC can be treated by surgery, chemotherapy, or radiotherapy^4^. There are different forms of radiation therapy, including 3D conformal radiation therapy, intensity-modulated radiation therapy, particle beam therapy and brachytherapy, which are commonly used in the treatments of cancers of the head and neck. Moreover, the expression of EBV latent proteins within undifferentiated nasopharyngeal carcinoma can be potentially exploited for immune-based therapies^5,6^.

Radiation therapy is a conventional method to stop cancer cells from growing or kill them altogether with high energy X-rays. As early as the early 1990s, the radical radiotherapy (RT) for treatment of NPC used two-dimensional RT, which was developed into three-dimensional conformal RT. With the development of technology, intensity-modulated radiotherapy (IMRT) is adopted in radiotherapy for NPC. Considering that IMRT can guarantee high local and regional control at increased toxicity rates, IMRT is gradually becoming the standard radiotherapy method for NPC. A retrospective study of IMRT for the treatment of NPC by Lai et al., compared with 2D-RT, the local tumor control rate of patients with NPC treated by IMRT was significantly improved, especially for the cases with stage T1 cancer (5-year local no recurrence survival rate was 100% vs 94.4%, P = 0.016)^7^. In the 1960s and 1970s, new combinations of chemotherapeutic agents using a variety of different mechanisms of action began to be proposed in clinical practice. Almost all RT, combined with chemotherapy, have achieved gratifying results, 2–5 years local control more than 90%. Chemotherapy, though widely used, the improvement of the remote control is not satisfactory. 2 years of distant metastasis rate is between 10–15%, 4 years distant metastasis rate is as high as 32%^8^

Although radiotherapy, or chemotherapy treatments have some effect on early-stage nasopharyngeal carcinoma, the outcome of patients with advanced NPC diagnosis is still far from expectation, with the median survival of only 12 months^9,10^. Therefore, a deeper understanding of the key biomarkers and molecular mechanisms of NPC progress could potentially lend insights to the therapeutic development of NPC. Accumulating researchers have identified many genetic and epigenetic aberrations in NPC, such as the mutation of ARID1A, TP53, PIN3CA, and others^11–13^. Intensity-modulated radiation therapy, simultaneous radiotherapy and chemotherapy are used in standard of care. However, the overall survival rate of NPC patients remains to be improved. Therefore, exploring the molecular mechanism of its dynamic development is of great importance to reduce the recurrence and metastasis rate. Gene chip technology and bioinformatics have achieved significant success in identifying tumor-associated genes and the underlying etiological mechanism. However, the microarray is characterized by large volume, high noise levels, small sample size and multiple data dimension^14^. Currently, there is no solution for microarray data processing and analysis that could resolve those aspects. Researches have shown that the control of type I error is critical to screen differentially expressed genes from expression profile data^15,16^. In this study, our ingenious UGM provides practical solution to some of the most common problems in microarray data analysis, especially the multiple validation of differential expressions, which warrants further validation for the application in the screening and identification of key biomarkers for NPC.

## Methods

### New method for screening differentially expressed genes

For the comparison of single gene difference, P value less than 0.05 is usually considered as statistical significance. However, there is still 5% of probability that this hypothesis is wrong. When 10,000 genes in two (group) samples are tested using the same test method, 500 (10,000*0.05 = 500) genes could be misestimated. After the 1950s, with the development of gene chip technology and large amount of data generated thereof, multiple hypothesis testing becomes widely used and increasing efforts have been made to address its problem. Table 1 illustrates the results of multiple hypothesis testing.

**Table 1:** Results of multiple hypothesis testing.

	Declared non-significant	Declared significant	Total
True null hypotheses	U	V	m0
Non-true null hypotheses	T	S	m1(m-m0)
Total	W(m-R)	R	M

m is the number of hypothesis tests, and the number of genes in gene chip data. m0 is the true and m1 is the false null hypothesis, where m1 = m-m0. After testing the m (null) hypothesis, it is declared that R is significant, and W (W = m-R) is non-significant null hypothesis. U, T, V, and S represent the summation of the judgment of samples in multiple comparisons. According to the judgment rules, m (null) hypotheses were divided into four parts, which are U, V, T and S, where U and S were the correct tests. V and T represented the number of type I and II error tests in m(null) hypotheses.

FDR (False Discovery Rate) can be calculated from Table 1, which represents the percentage of test results that reject the true null hypothesis in the sample. In 1995, Benjamin developed the FDR error control method^17^. FDR control method corrects the type I error in multiple hypothesis testing. FDR is a relatively conservative comparison method, and has greater power than FWER. FDR is outlined as follows

1 $$\mathrm{FDR}=\left\{\begin{array}{l}\mathrm{E}\left(\frac{\mathrm{V}}{\mathrm{S}+\mathrm{V}}\right)=\mathrm{E}\left(\frac{\mathrm{V}}{\mathrm{R}}\right)\mathrm{R}\ne 0\\ 0 \quad \mathrm{ R}=0\end{array}\right.$$

The evaluation of m0 is the most critical step in FDR program. The exactitude of m0 is key for the screening of DEGs, FDR control processes and testing capabilities. Our Unit Gamma Measurement (UGM) is a modified FDR control process with improved estimation of m0.

**Figure 1:**
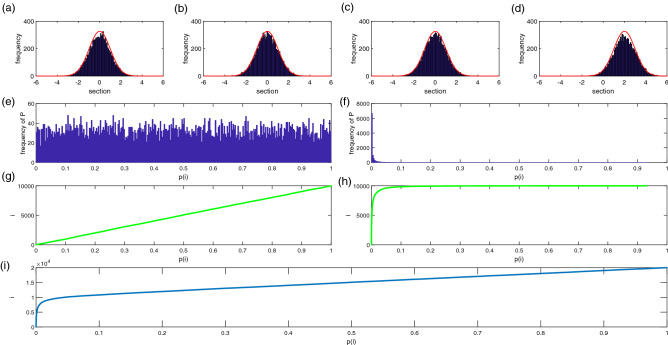
Relationship between P(i) and its frequency. (**a**) Sample 1 is randomly generated by N (μ1, σ1^2). (**b**) Sample 2 is randomly generated by N (μ2, σ2 ^ 2),${\mu }_{1}={\mu }_{2}$, and ${\sigma }_{1}={\sigma }_{2}$. (**c**) Sample 3 is randomly generated by N (μ3, σ3 ^ 2). (**d**) Sample 4 is randomly generated by N (μ4, σ4 ^ 2),${\mu }_{3}\ne {\mu }_{4}$, and ${\sigma }_{3}={\sigma }_{4}$. (**e**) Frequency distribution of P(i) in hypothesis testing using samples 1 and 2. At this time, H0 is true, and the calculated P value is evenly distributed between 0 and 1. (**f**) Frequency distribution of P (i) hypothesis testing of sample 3 and 4. H0 is false, and most of the calculated P value is distributed between 0 and 0.05. (**g**) P (i) vs i when samples 1 and 2 are used for hypothesis testing. The P-value accumulation curve is close to a straight line, which passes through two points (0, 0) and (0, m). (**h**) P(i) vs i when samples 3 and 4 are used for hypothesis testing. When the P value is small, the cumulative P value quickly reaches m. (**i**) P (i) vs i sample 1 and 2, sample 3 and 4 are paired for hypothesis testing, respectively. When the P value is very small, the accumulation of the P value rises quickly. When the P value is greater than some value (for example, 0.05), the accumulation curve of the P value approaches a straight line. The picture was drawn using R programming language (https://www.r-project.org/, v4.0.0).

Figure 1 shows that P value is a very regular nature in the ideal state. If the number of genes is m, and the ratio of the number of non-differentiated genes is ${\pi }_{0}$, the number of non-differentiated genes is ${m}_{0}=m*{\pi }_{0}$. Assuming that there is a value $\gamma$, which all differentil expression of gene test P values are distributed in $(0,\gamma )$. In this case, the genes distributed in $\left(\gamma ,1\right)$ should be all non-differentially expressed genes. Within this region, the number of non-differentially expressed genes in unit gamma length is $\underset{1\le i\le m}{\mathrm{min}}\{i:{P}_{i}^{*}\ge \gamma \}*\frac{\gamma }{1-\gamma }$. Therefore, the number of genes distributed in $(0,\gamma )$ should theoretically be the sum of all the differentially expressed genes and $\underset{1\le i\le m}{\mathrm{min}}\{i:{P}_{i}^{*}\ge \gamma \}*\frac{\gamma }{1-\gamma }$, i.e., the number of genes in $(0,\gamma )$ is $m-{m}_{0}+\underset{1\le i\le m}{\mathrm{min}}\{i:{P}_{i}^{*}\ge \gamma \}*\frac{\gamma }{1-\gamma }$. The number of non-differentially expressed genes in the multi-gammas is calculated to avoid the effect of random error.

The key of this algorithm is to evaluate ${m}_{0}$. Letting ${H}_{01},{H}_{02},{H}_{03},\dots ,{and H}_{0m}$ to be null hypothesis (genes). Correspondingly, the P-values of independent hypothesis tests are ${P}_{1},{P}_{2},{P}_{3},\dots ,{and P}_{m}$. The level of significance is $\mathrm{\alpha }$. UGM process is presented as follows:

Letting ${H}_{01},{H}_{02},{H}_{03},\dots ,{and H}_{0m}$ to be the tested null hypotheses. Using single test method to test each event and getting P values ${P}_{1},{P}_{2},{P}_{3},\dots ,{and P}_{m}$, and sorting p values ${P}_{1}^{*},{P}_{2}^{*},{P}_{3}^{*},\dots ,{and P}_{m}^{*}$.

Selecting the appropriate cutoff gamma to qualitatively divide the P value. Gamma should be greater than the level of significance. Gamma can be appropriately increased when there are lots of genes. Calculating the number of genes distributed in $\left(0,\gamma \right),\left(\gamma ,2\gamma \right),\dots ,\mathrm{and }(n*\gamma ,\left(n+1\right)*\gamma )$.$\left(n+2\right)*\gamma$ is greater than 1. We define $Pre\_\gamma$ and $Lat\_\gamma (k)$ as follows:

2 $$\left\{\begin{array}{l}Pr{e}_{\gamma }=\underset{1\le i\le m}{\mathrm{max}}\{i:{P}_{i}^{*}\le \gamma \}\\ La{t}_{\gamma \left(k\right)}=\underset{1\le i\le m}{\mathrm{max}}\left\{i:{P}_{i}^{*}\le k*\gamma \right\}k=\mathrm{1,2},3,\dots ,n\end{array}\right.$$

Estimate $m-{m}_{0}$. Estimation is calculated as follows:

3 $$m-{m}_{0}={\widehat{m}}_{1}=Pre\_\gamma -\sum_{i=1}^{n}{\tau }_{i}*Lat\_\gamma (i)$$

${\tau }_{i}$ is weight coefficient, which formula is as follows:

4 $${\tau }_{i}=\frac{1}{Lat\_\gamma (i)*{\sum }_{j=1}^{n}\frac{1}{Lat\_\gamma (i)}}$$

Getting $${\widehat{m}}_{0}$$

5 $${\widehat{\mathrm{m}}}_{0}=\mathrm{m}-{\widehat{\mathrm{m}}}_{1}$$

Adjust$$\mathrm{ing }{\mathrm{P}}_{\mathrm{i}}^{*}$$ by $${\mathrm{P}}_{\mathrm{i}}^{*}=\underset{\mathrm{i}\le \mathrm{k}\le \mathrm{m}}{\mathrm{min}}\{\mathrm{min}\{\frac{\widehat{{\mathrm{m}}_{0}}}{\mathrm{k}}*{\mathrm{P}}_{\mathrm{k}}^{*},1\}\}$$.

### Affymetrix microarray data

Three datasets were selected in the NCBI\GEO (Gene Expression Omnibus) database (https://www.ncbi.nlm.nih.gov/geo/) to identify DEGs in NPC with UGM. Three NPC datasets, namely GES64634, GSE12452 and GSE34573, were chosen from GEO datasets (Table 2). The GSE64634, GSE12452 and GSE34573 datasets were based on the GPL570 platforms ([HG-U133_Plus_2] Affymetrix Human Genome U133 Plus 2.0 Array, with 22,283 probes/genes). Among them, the GSE64634 dataset included 12 nasopharyngeal carcinomas tissue and 4 normal healthy nasopharyngeal tissue specimens. The GSE12452 dataset included 31 nasopharyngeal carcinomas and ten normal healthy nasopharyngeal tissue specimens. The GSE34573 dataset included 16 nasopharyngeal carcinomas and four normal healthy nasopharyngeal tissue specimens.

**Table 2:** Basic information of three NPC datasets.

References	Accession ID	Platform	Number of genes	Number of normal nasopharyngeal tissue samples	Number of NPC tissue samples
Bo et al.^18^	GSE64634	GPL570	12,625	4	12
Hsu et al.^19^	GSE12452	GPL570	12,625	10	31
Hu et al.^20^	GSE34573	GPL570	12,625	4	16

### GEO data treating and DEGs screening

The raw data of the three datasets downloaded from GEO datasets were processed by the UGM method to identify genes that are differentially expressed between NPC tissues and normal nasal tissues. In this process, we used the online tool GEO2R (http://www.ncbi.nlm.nih.gov/geo/geo2r) to calculate the two-sample t-test, and chose the calculated p value as the basis of UGM method, which was conducted using the R programming language (https://www.r-project.org/, v4.0.0). Criteria for DEGs screening were the adjustment of P value by UGM method less than 0.05 and |log FC (fold change)|≥ 2.0. Further, we used online tool Venny (http://bioinfogp.cnb.csic.es/tools/venny/index.html, v2.1.0) to identify DEGs.

### Gene function enrichment and annotation

The Screened DEGs were analyzed by the Database for Annotation, Visualization and Integrated Discovery (DAVID) online software (https://david.ncifcrf.gov, v6.8). This article used the Gene Ontology database (http://geneontology.org/) to annotate biological functions of DEGs.

### Hub gene disease association and process-focused annotation

In this process we used NetWorkAnalyst online software (https://www.networkanalyst.ca/, 3.0) to construct a disease network of DEGs. In NetWorkAnalyst, we set the number of subnetwork nodes to be more than 3 (nodes count ≥ 3), and counted the number of adjacent nodes in the interaction network and screened out key node genes that are connected with NPC disease. What’s more, combining KEGG signal pathway and GO enrichment analysis results, we used QuickGO (https://www.ebi.ac.uk/QuickGO/) to conduct an ancestor chart analysis of DEGs^21^.

### Consent for publication

All authors approved the manuscript and gave their consent for publication.

## Result

### Identification of DEGs with UGM

**Figure 2:**
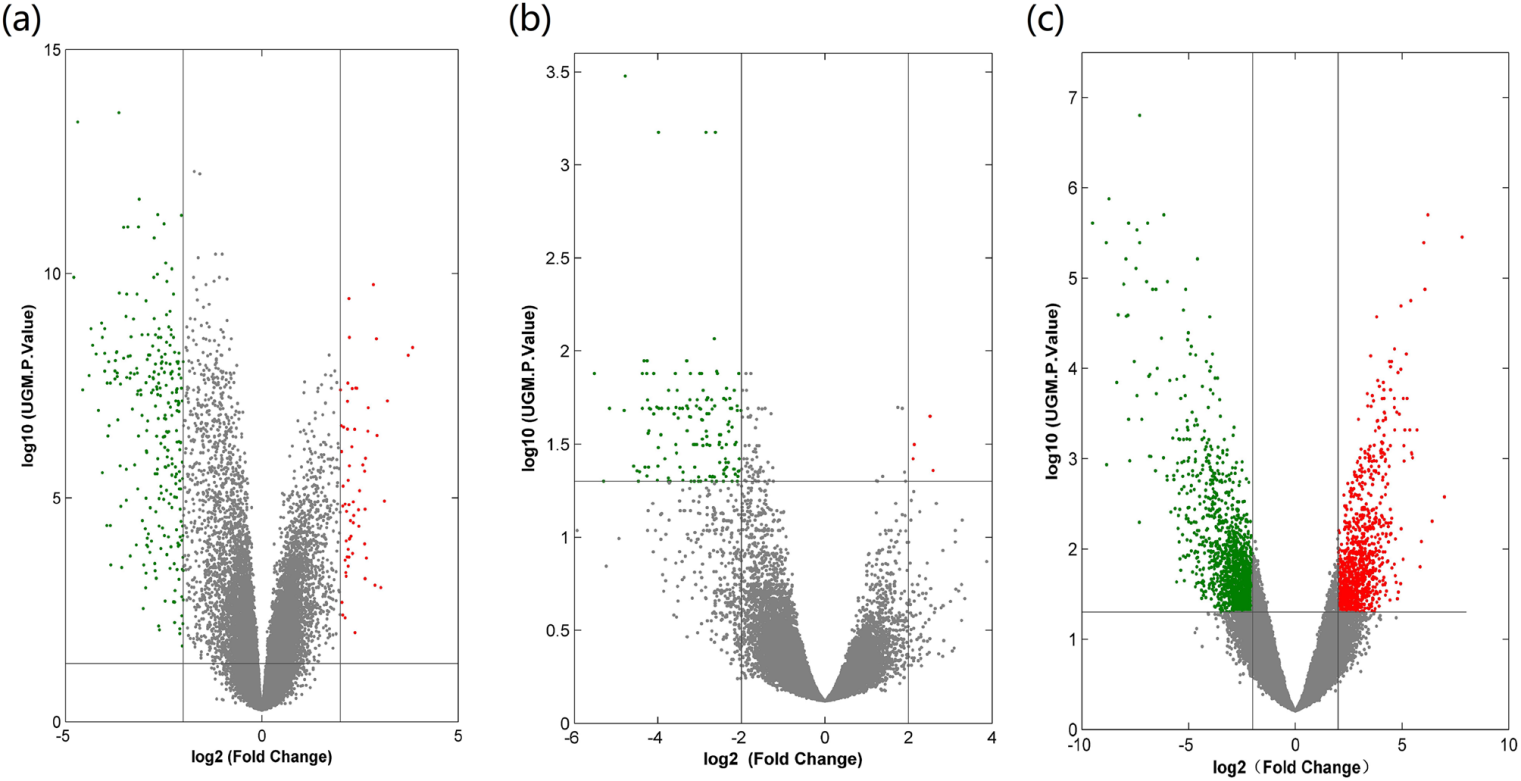
The volcano plots of DEGs in GSE12452 (**a**), GSE64634 (**b**) and GSE34573 (**c**) microarrays. The genes marked in red are upregulated genes, green marked are downregulated genes, and the gray represents non-differential expression genes at the cutoff P value < 0.05 and |log FC|> 2.0. The picture was drawn using online tool Venny (http://bioinfogp.cnb.csic.es/tools/venny/index.html, v2.1.0).

**Figure 3:**
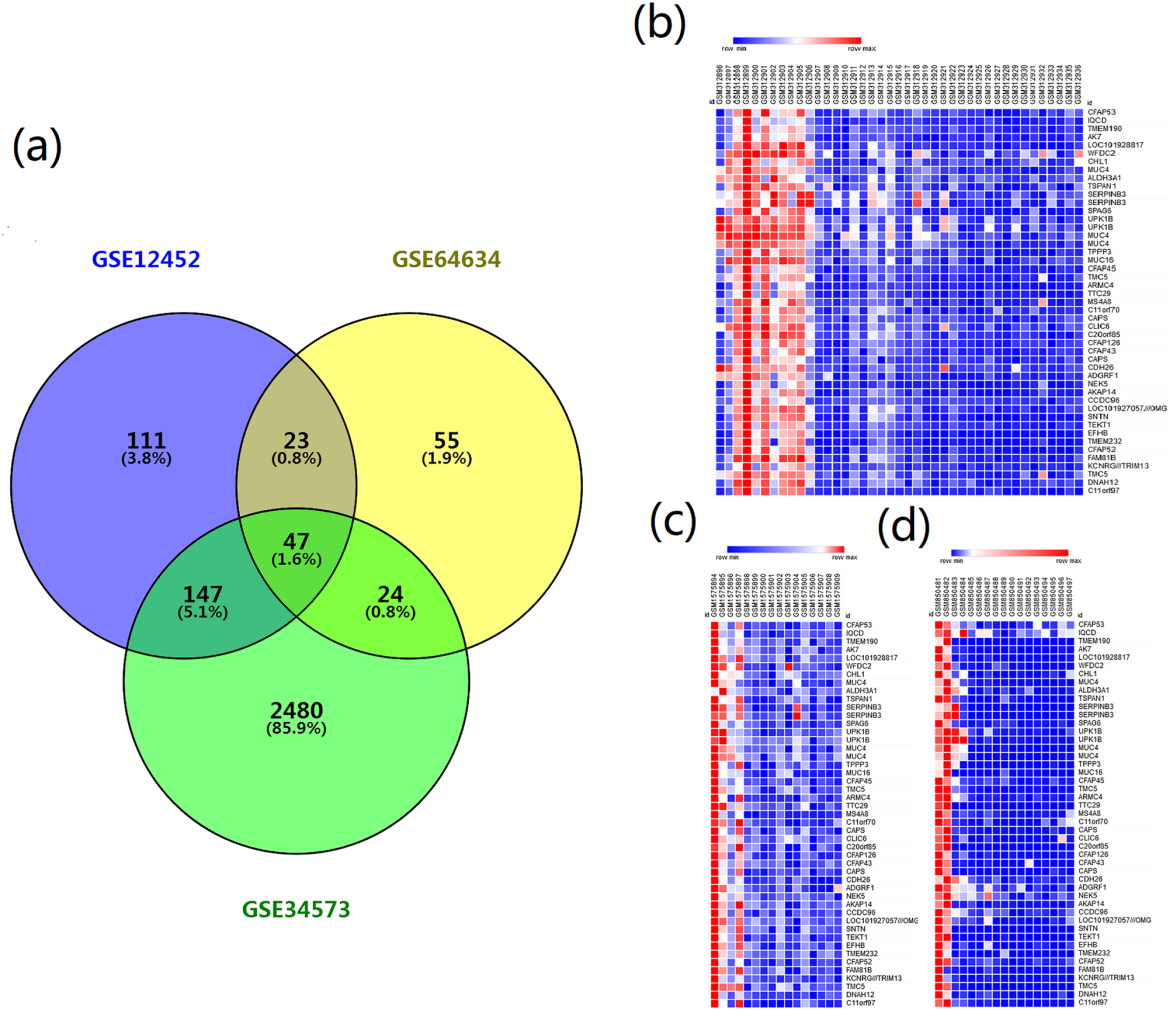
Heat maps and venn diagram of most significant DEGs. (**a**) Venn diagram of DEGs from intersection in 3 GSE datasets. There are 69 intersecting DEGs between GSE12452 and GSE64634, 71 DEGs between GSE64634 and GSE34573, and 194 DEGs between GSE12452 and GSE34573, and 47 DEGs are presented in all GSE datasets. (**b**) Heat map of 47 DEGs in GSE12452, (**c**) GSE64634 and (**c**) GSE34573 datasets. The red marked block indicates the high-level of gene expression and the blue indicated low-level expression. The picture was drawn using R programming language (https://www.r-project.org/, v4.0.0).

After the standardization of the microarray and identification DEGs by the UGM and |log FC (fold change)|> 2.0, a total of 328 DEGs in GSE12452 were identified, including 266 down-regulated genes and 62 up-regulated genes (Fig. 2a); GSE64634 has 149 DEGs, consisting of 145 down-regulated genes and 4 up-regulated genes (Fig. 2b); GSE34573 has 2698 DEGs, comprising1664 down-regulated genes and 1634 up-regulated genes (Fig. 2c). These 3 data sets have 47 DEGs overlapped and were sequenced according to the average log2FC value and analyzed by Rank analysis. The expression of DEGs was presented as heat map in Fig. 3.

### Functional enrichment and pathway analysis of the DEGs

**Figure 4:**
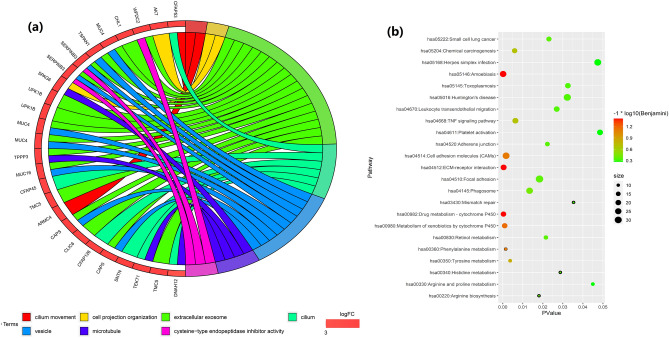
GO and KEGG pathway enhancement analysis DEGs in NPS and normal tissue. (**a**) GO function enhancement analysis DEGs in NPS and normal tissue. Log2FC, log2 (fold change). GO Terms, GO functional notes. (**b**) KEGG pathway enhancement analysis of DEGs between NPC and normal tissue. Log10 (Benjamin), log10 (the value of Benjamin adjustment to P value). Gene counts enriched in the pathway are presented proportional to the size of bubble. Enriched KEGG pathway includes amoebiasis, drug metabolism—cytochrome P450, ECM-receptor interaction, metabolism of xenobiotics by cytochrome P450, phenylalanine metabolism, cell adhesion molecules, tyrosine metabolism, chemical carcinogenesis, TNF signaling pathway, phagosome, arginine biosynthesis, focal adhesion, retinol metabolism, adherens junction, small cell lung cancer, leukocyte transendothelial migration, histidine metabolism, huntington disease, huntington disease, mismatch repair, arginine and proline metabolism, herpes simplex virus 1 infection and platelet activation. All of them can be obtained from https://www.kegg.jp/kegg/pathway.html. The picture was drawn using R programming language (https://www.r-project.org/, v4.0.0).

To analyze the biological classification of DEGs, functional and pathway enrichment analyses were performed using DAVID. GO analysis results showed that the cell component (CC) in those DEGs over represented are cilium, vesicle, microtubule and extracellular exosome. The enriched biological processes (BP) are cilium movement and cell projection organization. At molecular function (MF) level, cysteine-type endopeptidase inhibitor activity was enriched (Table 3 and Fig. 4a). KEGG pathway analysis demonstrated that these DEGs were enriched in ECM-receptor interaction, cell adhesion molecules (CAMs), chemical carcinogenesis, TNF signaling pathway, small cell lung cancer, mismatch repair, phagosome, etc. (Fig. 4b).

**Table 3:** GO function enhancement analysis of DEGs in NPC samples.

Category	GO ID	Description	Count in gene set	P.Value
CC	GO:0005929	Cilium	6	< 0.05
CC	GO:0031982	Vesicle	5	< 0.05
CC	GO:0005874	Microtubule	4	< 0.05
CC	GO:0070062	Extracellular exosome	11	< 0.05
BP	GO:0003341	Cilium movement	2	< 0.05
MF	GO:0004869	Cysteine-type endopeptidase inhibitor activity	2	< 0.05
BP	GO:0030030	Cell projection organization	2	< 0.05

*CC* cell component, *BP* biological processes, *MF* molecular function.

### Hub gene disease association and process-focused annotation on 47 DEGs analysis

**Figure 5:**
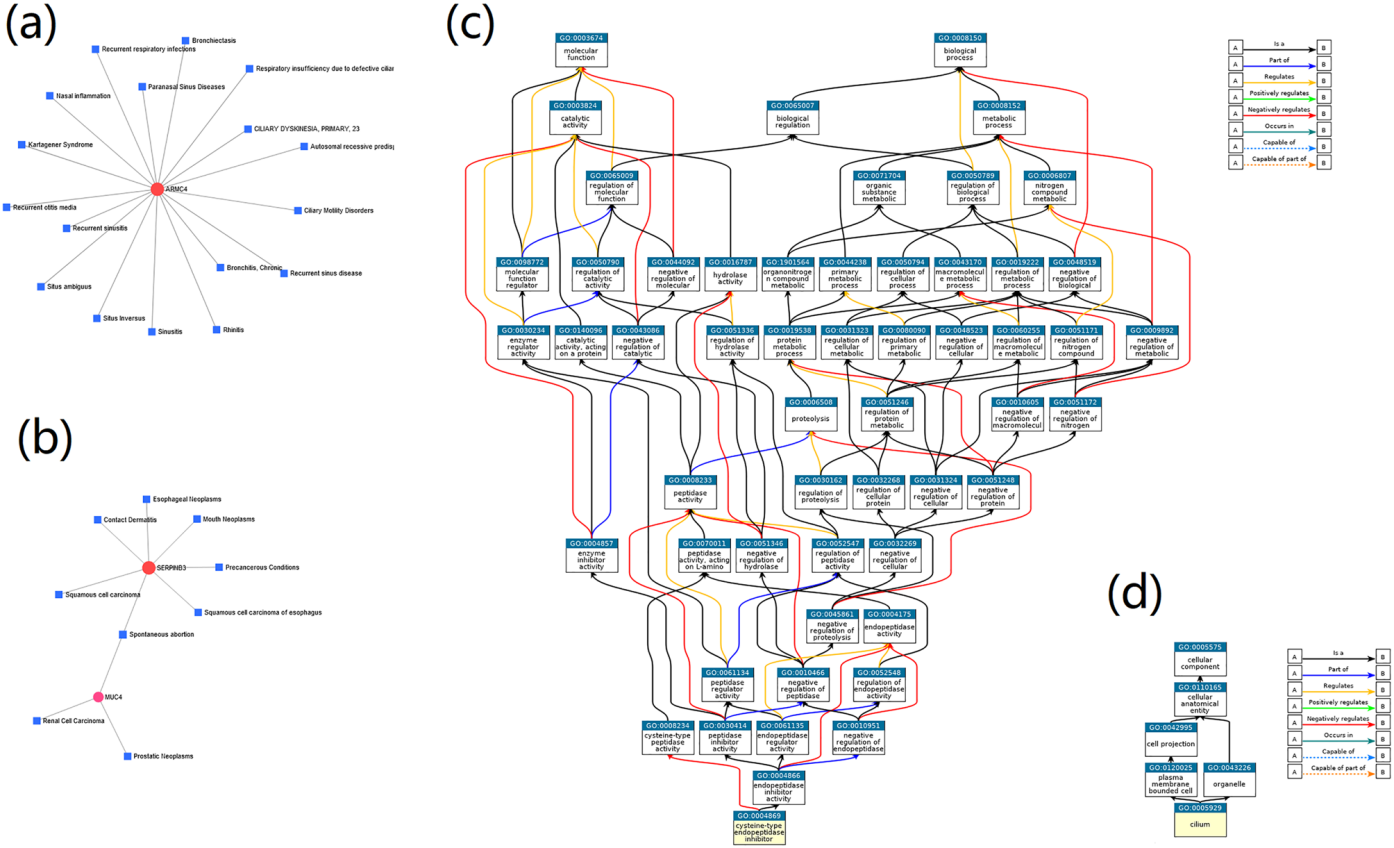
Hubs of DEGs with strong association with diseases and the process-focused annotation. The largest (**a**) and second largest (**b**) sub-networks. The red circle area represents the most significantly disease-associated genes (hub genes), and the blue square area represents genes related to the hub genes. QuickGO term (GO: 0,004,869 ‘cysteine-type endopeptidase inhibitor activity’(**c**) and GO: 0,005,929 ‘cilium’ (**d**)) ancestor chart. Currently, eight relationship types are described in Huntley^45^. Briefly, ‘is a’ presents a subclass of its parent, ‘part of’ stands for part of the parent term, ‘regulates’ is a process that modulates its parent process, and ‘positively regulates’ and ‘negatively regulates’ enhance and decrease the modulation of a parent process term, respectively. The fig (**a**,**b**) were drawn using online tool NetWorkAnalyst online software (https://www.networkanalyst.ca/, 3.0), and fig (**c**,**d**) were drawn using online tool Venny QuickGO (https://www.ebi.ac.uk/QuickGO/).

The online analysis software NetWorkAnalyst3.0 was employed to generate a network composed of gene hubs associated with diseases using the 47 overlapping DEGs. The significant genes from previous analysis were mapped to the corresponding molecular DisGeNET database. The procedures typically produced several sub-networks that were shown in Fig. 5a,b. The diseases strongly associated with 47 DEGs were mainly Kartagener syndrome, paranasal sinus diseases, rhinitis, sinusitis, recurrent otitis media, nasal inflammation, and respiratory insufficiency due to defective ciliary clearance, recurrent respiratory infections, primary ciliary dyskinesia (23), autosomal recessive predisposition, and recurrent sinus disease. QuickGO ancestor chart provides functional ontologies for GO: 0004869 ‘cysteine-type endopeptidase inhibitor activity’ and GO:0005929 ‘cilium’ (Fig. 5c,d).

### Analysis of the correlation between key genes and NPC

**Figure 6:**
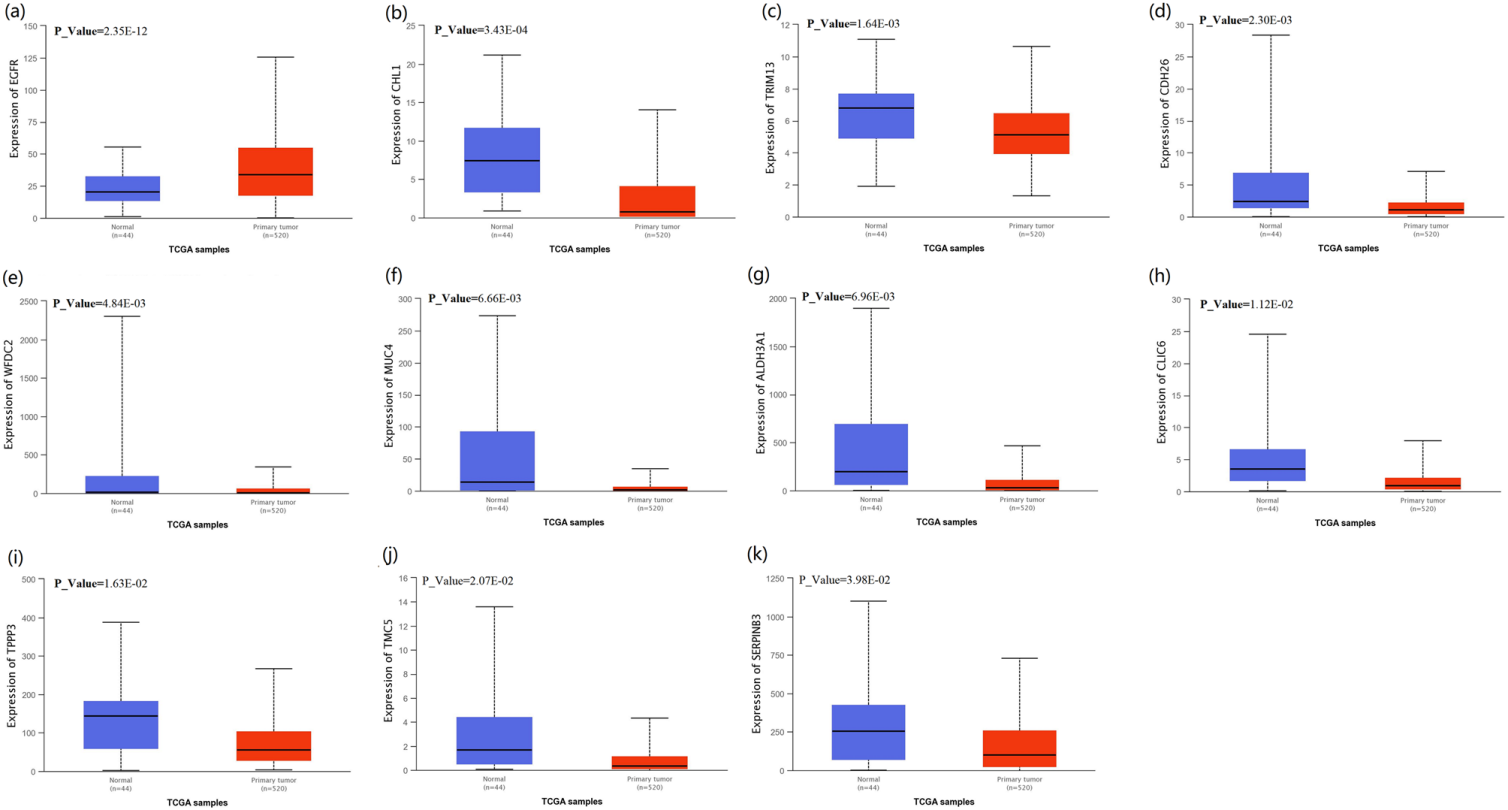
The significant up-regulation or down-regulation in head and neck squamous cell carcinoma were compared with normal samples. Expression of (**a**) ECFR, (**b**) CHL1, (**c**) TRIM13, (**d**) CDH26, (**e**) WFDC2, (**f**) MUC4, (**g**) ALDH3A1, (**h**) CLIC6, (**i**) TPPP3, (**j**) TMC5 and (k) SERPINB3 in TCGA samples. The picture was drawn using R programming language (https://www.r-project.org/, v4.0.0).

**Figure 7:**
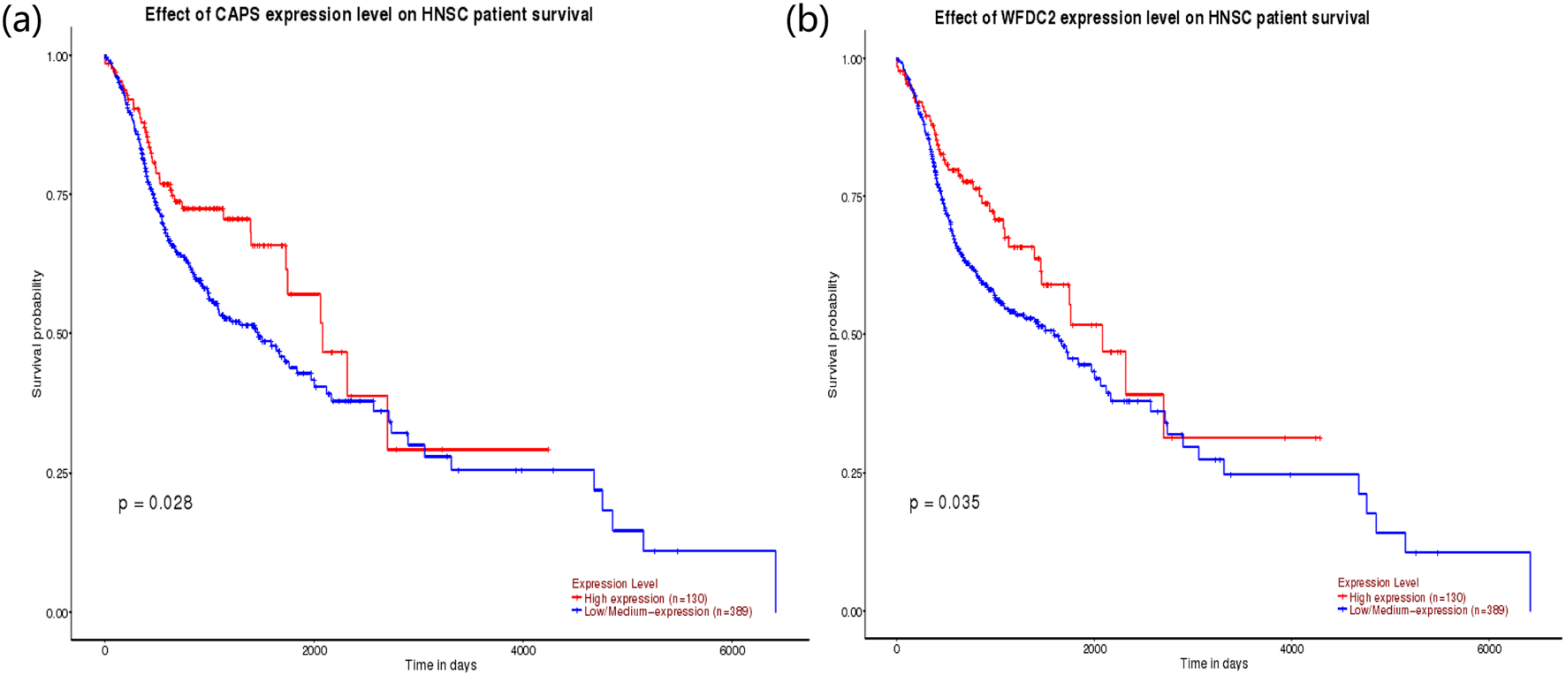
Kaplan Meier’s survival analysis. Effect of (**a**) CAPS and (**b**) WFDC2 expression level on head and neck squamous cell carcinoma patient survival. Down-regulation of CAPS and WFDC2 can prolongation of the overall survival periods in the patients. The picture was drawn using R programming language (https://www.r-project.org/, v4.0.0).

We download the gene expression information and clinical information of the data related to NPC from the TCGA database (https://portal.gdc.cancer.gov/). A total of 564 cases (44 normal cases and 520 NPC patients) were selected. We used the TCGA database to verify the bioinformatics findings of the 47 differentially expressed genes screened, and found that, among the 47 screened genes, the significant up-regulation or down-regulation of EGFR, CHL1, TRIM13, CDH26, WFDC2, MUC4, ALDH3A1, CLIC6, TPPP3, TMC5 and SERPINB3 in head and neck squamous cell carcinoma were compared with normal samples (Fig. 6). In addition, consistent with the expression of CAPS and WFDC2 in nasopharyngeal carcinoma, Kaplan Meier’s survival analysis exhibited the remarkable prolongation of the overall survival periods in the patients with low CAPS and WFDC2 (Fig. 7).

## Discussion

Microarray technology could provide abundant information on gene expression under certain circumstances from the hybridization signal^22,23^. The ease of data acquisition, high through-put, large data volume, and small sample size have made it a widely applied tool in biological inquisition. However, the high levels noise and multiple data dimensions leave the current data processing an outstanding problem.

Recently, gene chips were mainly used in biological researches to differentiate subtypes of diseases and predict the prognosis of patients. Unsupervised algorithms, such as cluster analysis methods, are most commonly applied for microarray analysis to identify sub-class of diseases. Supervised algorithms such as discriminant analysis methods, artificial neural network models and other methods, were usually used to differentiate the degrees of disease prognosis^24,25^. However, the application of the analysis is preceded by the reduction of the data dimensions and false positives when selecting DEGs among different comparing groups. The purpose of this paper is to provide UGM, a practical solution to the most common practical problems in microarray data analysis, especially the multiple validation of differential expressions, which could assist in the screening and identification of key biomarkers for NPC.

For the past decades, data analysis methods for gene expression profiles have attracted extensive interests in the community of biological and medical statistics. The key to screen DEGs from gene expression profile data is to reduce type I error and ensure a high screening efficiency. A variety of methods have been proposed to address these problems. It is well-recognized that the expected percentage of the null hypothesis that is wrongly rejected is a meaningful indicator in multiple comparisons, but not the probability of error detection. Based on this assumption, Benjamin and Hochberg developed the FDR control program, which was a groundbreaking achievement. It has been widely used in processing large-scale data following the seminal paper by Benjamin and Hochberg in 1995. Subsequent improvements and extensions of Benjamin and Hochberg method have been proposed^26–32^. In recent years, the subject interests have been focused on the evaluation of m0, which is critical for the screening of DGEs, FDR control and gauging testing capabilities. However, we found that the estimation method proposed in this process is compromised. Although the average estimated values are very close to the true value over the course of iterations, it is still far from the standard deviation. This introduces large amount of random errors, thus rendering inaccurate results. Therefore, we proposed UGM, a new FDR control process based on m0 estimation. In the present study, the identification of critically and differentially expressed genes (DEGs) in NPC with UGM and subsequent functional analysis of DEGs demonstrated the effectiveness of this tool in inquiring the molecular mechanism of NPC development. Three mRNA expression profiling of NPC in GEO dataset were selected as input of UGM. A total of 47 DEGs were screened for further analysis of biological functions. Among these DEGs, the Armadillo Repeat Containing 4 (ARMC4) was significantly up-regulated in NPC tissues. This result is consistent with the results reported by Hjeij R^33^. ARMC4 may inhibit the proliferation and division of NPC cells by participating in the Cilium pathway, Coiled coil pathway and repeat: ARM 6 pathway, etc. Many diseases are strongly associated with ARMC4, such as Kartagener syndrome, paranasal sinus diseases, rhinitis, sinusitis, recurrent otitis media, nasal inflammation, and respiratory insufficiency due to defective ciliary clearance, recurrent respiratory infections, primary ciliary dyskinesia(23), autosomal recessive predisposition, and recurrent sinus disease, all of which have direct or indirect relations to NPC. The Serpin Family B Member 3 (SERPINB3) and Mucin 4, cell Surface Associated were also significantly up-regulated in NPC tissues, and they may inhibit the normal expression of NPC cells by participating in polymorphism pathway and sequence variation pathway^34,35^. Diseases, including prostatic neoplasms, squamous cell carcinoma, esophageal neoplasms, mouth neoplasms, precancerous conditions and squamous cell carcinoma of esophagus had a strong connection with SERPINB3 and UMC4.

Further, we conducted functional enrichment and pathway analysis of the screened DEGs. The DEGs functions were mainly enriched in biological processes such as extracellular exosome, tripartite motif-containing protein 13, sequence variant, polymorphism, cysteine-type endopeptidase inhibitor activity, cytoskeletal regulation and vesicle trafficking signaling pathway etc.^36–39^. These overrepresented biological processes were cell cycle, biological immunity, signaling, DNA repair, cytoplasmic transport, cell proliferation, migration and invasion. Noteworthily, 11 DEGs (11/47) were enriched in extracellular exosome, a channel for cells to excrete wastes. Currently, studies on exosomal material composition and transportation, signal transmission between cells and distribution in body fluids have uncovered various functions of exosomes. They are involved in all aspects of body's immune response, antigen presentation, cell migration, cell differentiation, tumor invasion, etc.^31^. Moreover, tumor-derived exosomes mediate the exchange of genetic information between tumor cells and basal cells, thus leading to the formation of new blood vessels, which facilitates tumor growth and invasion.

In addition, there were 3 (CHL1, TSPAN1 and CCDC19) out of 47 DEGs screened by UGM method were proven by many scholars to be used as molecular markers for NPC^40–44^. CHL1 is a neural recognition protein that may be involved in signal transduction pathways. Recently, several cell adhesion molecules, including L1, were shown to be involved in cancer growth and metastasis^40^. 3p26 has been reported to harbor a candidate gene for prostate cancer susceptibility in Finnish prostate cancer families; however, no mutations were detected in the coding part of CHL1^40,41^. Nevertheless, these reports suggest that plays a pivotal role in cancer development. Furthermore, functional study showed that ectopic expression of CHL1 in NPC cells dramatically inhibited their clonogenicity and migration as compared with parental NPC cells without CHL1 expression. Shilong Xiong found that real-time quantitative reverse transcription-PCR and in situ hybridization (ISH) techniques confirmed that TSPAN-1 and DPP10 genes had only 40.72% and 40.70% positive expression in NPC, but had high positive expression in chronic inflammation of nasopharyngeal mucosa^41^. The data suggested that TSPAN-1 might be the putative molecular markers of NPC. Zhen Liu found that CCDC19 was specifically expressed in the nasopharynx epithelium and its reduced expression is an unfavorable factor promoting NPC progression and poor prognosis^43,44^. CCDC19 was identified as a potential tumor suppressor in NPC pathogenesis due to its decreased expression in NPC patients and its inhibitory function in NPC cells. In addition, among the 47 differentially expressed genes we screened, significant up-regulation or down-regulation of 11 genes (EGFR, CHL1, TRIM13, et al.) expression were observed in the TCGA database. Kaplan Meier’s survival analysis exhibited the remarkable prolongation of the overall survival periods in the patients with low CAPS and WFDC2, which means that CHL1, CAPS and WFDC2, etc., might be the putative molecular markers of NPC.

In this article, we proposed a method for screening differentially expressed genes based on gene chip data, but we were also aware of its limitations that need to be further studied. Firstly, the UGM needs to be practiced and promoted. The UGM method can be used in NPC data to screen differentially expressed genes. However, it is necessary to further explore the application of this method to other tumor gene chip data, such as breast cancer, pancreatic cancer, prostate cancer, esophageal cancer, et al. The goal of UGM method research is to discover new and unknown hub genes (proto-oncogene or tumor suppressor gene) and determine the pathway in the cell, which requires more in-depth analysis and can withstand medical clinical practice tests. In addition, it is necessary to further study the medical mechanism of hub genes in tumor diseases. The algorithm proposed in this paper was based on geometric characteristics of multivariable statistical analysis. At the same time, the effectiveness of the algorithm also needs gene chip (microarray) data. The practice of tumor research shows that gene expression bears some relations to the tumor occurrence, evolution and metastasis. There are many kinds of cancer sample data. Taking the heterogeneity of gene expression into account, for multi-sample gene chips, we also need to explore the UGM method to screen the differentially expressed genes in normal group samples and different stages cancer group samples.

In summary, the key step in the FDR process is to estimate the number of non-differentially expressed genes. However, we found that the estimation method proposed in this process is not accurate enough. So we designed a new method to estimate the number of non-differentially expressed genes on the basis of previous researches. Three nasopharyngeal carcinoma chip dataset housed in public database were used to screen differentially expressed genes, with UGM as a verification of the accurate and robust UGM. Further, ARMC4, SERPINB3 and UMC4 were identified as the most significant DEGs, which implicate strong association with NPC in functional enrichment and pathway analysis. Due to limited experiment validation, our study warrants further investigations using clinical samples to verify the association of DEGs with nasopharyngeal carcinoma and reveal the underlying mechanisms.

## Data Availability

The gene chip data are available at https://www.ncbi.nlm.nih.gov/. The gene-disease association analysis is available at https://david.ncifcrf.gov, http://www.ncbi.nlm.nih.gov/geo/geo2r, http://bioinfogp.cnb.csic.es/tools/venny/index.html, https://www.networkanalyst.ca/, and https://www.ebi.ac.uk/QuickGO/. All data and materials are fully available without restriction.
